# Genome Editing Approaches in Flax (*Linum usitatissimum* L.): From Tools to Trait Improvement

**DOI:** 10.3390/ijms27136012

**Published:** 2026-07-04

**Authors:** Marta Podralska, Aleksandra Górska, Mariusz Kaczmarek

**Affiliations:** Institute of Natural Fibres and Medicinal Plants—National Research Institute, Wojska Polskiego 71B, 60-630 Poznań, Poland

**Keywords:** genome editing, CRISPR/Cas9, *Linum usitatissimum*, flax, functional genomics, base editing, prime editing, fiber quality

## Abstract

Genome editing, particularly CRISPR/Cas-based systems, has emerged as a key tool for functional genomics and trait improvement in flax (*Linum usitatissimum* L.), an important fiber and oilseed crop. This review focuses specifically on flax as an emerging target species and distinguishes experimentally validated applications from approaches adapted from model plants. Recent progress includes the characterization of endogenous U6 promoters, which improved guide RNA expression and contributed to enhanced genome editing performance under optimized conditions. Reported studies demonstrate efficient targeted mutagenesis in flax; however, editing outcomes remain strongly dependent on genotype, construct design, and regeneration capacity, and stable homozygous edited lines are still limited. Target genes include pathways involved in lignin and cellulose biosynthesis, fatty acid metabolism, and stress responses, influencing fiber quality, oil composition, and stress adaptation. Despite current bottlenecks such as low homologous recombination efficiency and regeneration constraints, base editing, prime editing, and multiplex CRISPR systems provide promising avenues for precision breeding in flax.

## 1. Introduction

Genome editing technologies—collectively referred to as “genetic scissors”—have emerged as transformative tools in modern plant biotechnology, enabling precise, targeted modifications of genomic sequences. Among the various genome editing systems, CRISPR/Cas9 has gained particular prominence due to its versatility, ease of design, and high specificity. Its performance continues to improve through optimization of experimental design, delivery methods, and species-specific factors, which may facilitate regulatory approval in some jurisdictions [1,2]. Other nuclease-based platforms, such as TALENs (Transcription Activator-Like Effector Nucleases) and ZFNs (Zinc Finger Nucleases), have also been effectively deployed in plant systems, though their broader utility is constrained by technical complexity and cost-intensive assembly processes [3,4].

In the context of agriculture, genetic engineering has long been regarded as a potent strategy for improving crop performance by introducing or modifying traits such as yield potential, abiotic stress tolerance, and resistance to pathogens [5,6]. Traditional breeding approaches, while historically successful, are inherently limited by long generation times, linkage drag, and the narrow genetic base of cross-compatible species. By contrast, genome editing offers the potential for rapid and precise improvement of specific traits through single- or multigene targeting, often without the incorporation of foreign DNA, which may reduce regulatory constraints in some jurisdictions [7,8].

The application of genome editing in crop species has opened new avenues for addressing the mounting challenges of global food security in the face of climate change, land degradation, and resource limitations. Key agronomic traits such as drought tolerance, nutrient use efficiency, flowering time regulation, and oil composition are now within reach of precise molecular control [9,10,11]. Numerous staple and industrial crops—including rice, maize, soybean, and tomato—have already benefited from genome editing research aimed at both trait discovery and commercial cultivar development [12,13,14].

Within this landscape, flax (*L. usitatissimum* L.) represents a strategically important, yet comparatively underutilized, target for genome editing. Flax is regarded as one of the oldest and most versatile cultivated crops, with archaeological evidence of its cultivation dating back to before 6000 BC. Owing to its dual role as a fiber and oilseed crop, flax has been extensively utilized in industrial, nutritional, and pharmacological applications [15]. Flaxseed is particularly rich in α-linolenic acid (ALA), lignans, and dietary fiber, supporting its classification as a functional food with well-documented nutritional benefits [16,17,18,19].

Despite these valuable attributes, flax has received relatively limited attention in comparison to major cereal crops in terms of genomic resources, transformation protocols, and trait mapping. However, recent advancements in whole-genome sequencing [20,21], transcriptomics, and *Agrobacterium*-mediated transformation have laid the groundwork for the application of CRISPR/Cas-based editing in flax, targeting key pathways related to oil composition, fiber quality, and stress resilience [20,21,22,23].

Beyond its traditional uses, flax also represents a valuable model for dual-purpose crop improvement, where both seed- and fiber-related traits can be simultaneously optimized through targeted genome editing. Its relatively small genome, short growing season, and responsiveness to controlled environmental conditions make it a practical candidate for functional genomics studies and rapid trait validation. Moreover, flax occupies a unique niche in sustainable agriculture due to its low input requirements and adaptability to marginal soils, making it particularly relevant under climate change scenarios where resource-efficient crops are increasingly prioritized. Genome editing in flax therefore not only has the potential to enhance economically important traits, such as oil quality, fiber strength, and disease resistance, but also to contribute to the development of more resilient and environmentally sustainable agro-ecosystems.

Unlike previous reviews focusing broadly on plant genome editing technologies, this review specifically addresses flax as an emerging target species for genome engineering. Particular attention is given to recent advances in flax genomics, endogenous promoter development, transformation systems, multiplex genome editing, and future precision breeding applications.

## 2. Genome Editing Tools

Genome editing tools are molecular technologies that enable precise modifications of genomic DNA, including insertion, deletion, or substitution of specific sequences at defined locations. They have significantly advanced research in functional genomics, plant biotechnology, and gene therapy.

First-generation genome editing tools include zinc finger nucleases (ZFNs), which are engineered chimeric proteins consisting of a DNA-binding zinc finger domain fused to the FokI endonuclease. ZFNs introduce site-specific double-strand breaks (DSBs), which are repaired by endogenous cellular pathways such as non-homologous end joining (NHEJ) or homology-directed repair (HDR), enabling targeted genetic modifications [24].

**Figure 1:**
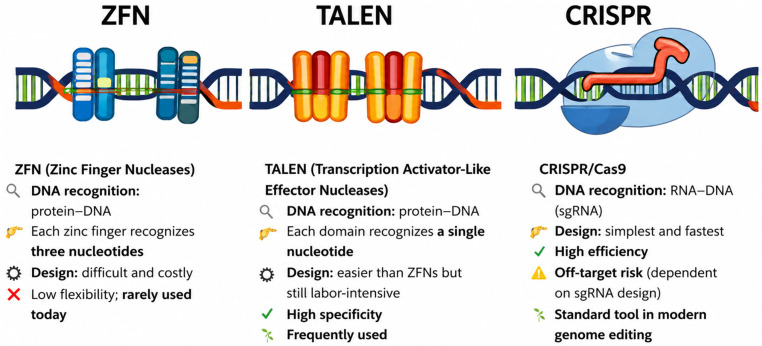
Comparison of genome editing tools.

Transcription activator-like effector nucleases (TALENs), developed subsequently, utilize customizable TALE DNA-binding domains derived from *Xanthomonas* species fused to the *FokI* nuclease. Compared to ZFNs, TALENs provide improved specificity and modularity and have been widely applied in both plant and animal systems [25]. A comparison of genome editing tools is shown in Figure 1.

## 3. CRISPR-Based Genome Engineering in Plants: Mechanisms, Delivery Technologies, and Emerging Applications

The adaptation of CRISPR-Cas systems from the adaptive immune machinery of bacteria and archaea has transformed genome engineering across biological disciplines, including plant biotechnology [2]. Among the available CRISPR platforms, CRISPR/Cas9 remains the most extensively utilized system owing to its simplicity, versatility, and high editing efficiency. The platform consists of the *Streptococcus pyogenes* Cas9 endonuclease and a single-guide RNA (sgRNA) containing a 20-nucleotide spacer sequence complementary to the target DNA region located upstream of a 5′-NGG-3′ protospacer adjacent motif (PAM). Following target recognition, the HNH and RuvC nuclease domains generate a site-specific double-strand break (DSB) approximately three nucleotides upstream of the PAM sequence [2]. The induced DSB is primarily repaired through non-homologous end joining (NHEJ), an error-prone pathway that frequently introduces insertions or deletions, thereby disrupting gene function and enabling efficient gene knockout. Alternatively, homology-directed repair (HDR) allows precise sequence replacement or insertion using homologous donor templates. However, HDR remains inefficient in most plant species due to limited activity during specific cell-cycle phases and competition with NHEJ. Consequently, considerable efforts have focused on improving HDR efficiency through donor-template amplification, manipulation of DNA-repair pathways, and cell-cycle synchronization strategies [25,26,27,28,29,30].

Successful genome editing depends not only on nuclease activity but also on efficient delivery of editing reagents. Conventional approaches include *Agrobacterium*-mediated transformation, particle bombardment, and polyethylene glycol (PEG)-mediated protoplast transfection. Although these methods have enabled routine genome editing in numerous crop species, their effectiveness is often restricted by genotype dependence, tissue-culture requirements, regeneration inefficiencies, and unintended transgene integration [31,32,33,34,35].

Strategies based on developmental regulators such as BABY BOOM (BBM), WUSCHEL2 (WUS2), and GRF4/GIF1 promote de novo meristem formation directly in transformed tissues, substantially improving regeneration efficiency and expanding applicability to recalcitrant genotypes. Closely related in planta genome-editing approaches bypass callus regeneration entirely by delivering CRISPR reagents directly into meristems, floral tissues, pollen, embryos, or axillary buds. Methods including Fast-TrACC, cut-dip-budding, meristem-targeted transformation, and in planta particle bombardment have demonstrated the feasibility of generating edited plants without conventional tissue culture, thereby reducing transformation time and genotype dependency [36,37,38,39].

Another rapidly expanding area is virus-induced genome editing (VIGE). Viral vectors such as *Tobacco rattle virus* (TRV) and *Barley stripe mosaic virus* (BSMV) provide systemic delivery of genome-editing components throughout plant tissues. The incorporation of mobile RNA elements further facilitates transport into meristematic regions, enabling heritable genome modifications without stable transgene integration. The emergence of compact nucleases, including Cas12f, CasΦ, and engineered TnpB variants, has further increased the potential of viral delivery systems by overcoming cargo-size limitations inherent to plant viruses [40,41,42,43,44].

Nanotechnology-based delivery platforms have also emerged as promising alternatives to conventional transformation methods. Carbon nanotubes, mesoporous silica nanoparticles, lipid nanoparticles, magnetic nanoparticles, and cell-penetrating peptides can transport DNA, RNA, or ribonucleoprotein (RNP) complexes across the plant cell wall while minimizing cellular damage. Although several studies have demonstrated efficient transient expression and DNA-free genome editing using these carriers, challenges associated with reproducibility, scalability, and species-specific responses still limit their broader application [45,46,47,48].

Particular attention has recently been directed toward DNA-free genome editing through direct delivery of CRISPR-Cas ribonucleoproteins. Compared with DNA-based transformation systems, RNP-mediated editing eliminates the risk of transgene integration and reduces off-target activity associated with prolonged nuclease expression. Electroporation-based approaches and biolistic RNP delivery have already demonstrated efficient editing in several economically important crop species, highlighting their potential for regulatory-friendly crop improvement [49,50].

The increasing complexity of crop traits has additionally stimulated the development of multiplex genome-editing strategies. Polycistronic guide RNA expression systems, including tRNA-processing, Csy4-mediated, and single-transcript-unit architectures, enable the simultaneous modification of multiple loci and facilitate engineering of complex polygenic traits, particularly in polyploid crops [51,52].

Collectively, these advances are reshaping the delivery landscape of plant genome editing and are expected to facilitate broader adoption of CRISPR technologies across diverse crop species. Tissue culture-independent systems, in planta editing strategies, viral vectors, nanocarriers, compact nucleases, and RNP-based delivery platforms represent some of the most promising innovations for next-generation crop improvement and precision breeding [34].

An overview of genome editing delivery methods in plants is provided in Table 1.

The advantages of CRISPR/Cas9 over earlier genome editing platforms such as ZFNs and TALENs are clear: it is cost-effective, scalable, and programmable via simple gRNA design [92]. Editing efficiencies are high—often exceeding 80% biallelic or homozygous mutations in T_0_ plants when optimized promoters and delivery systems are used [11].

While CRISPR/Cas9 remains the most widely used genome-editing platform in plants, continued efforts to expand targeting flexibility, improve precision, and facilitate delivery have led to the development of numerous alternative CRISPR systems and next-generation genome-engineering technologies.

In addition to Cas9-derived systems, several alternative CRISPR effectors have further expanded the genome engineering toolbox available for plant biotechnology. Cas12a (Cpf1) recognizes T-rich PAM sequences (TTTV) and generates staggered double-strand breaks, making it particularly useful for multiplex genome editing and targeting genomic regions that are less accessible to SpCas9. Cas12a-mediated genome editing has been successfully demonstrated in rice, maize, wheat, and *Arabidopsis*, where efficient targeted mutagenesis and multiplex editing of agronomically important genes were achieved [93,94,95]. Cas12b has also demonstrated efficient genome editing activity in rice and *Arabidopsis*, exhibiting high target specificity and reduced off-target effects compared with conventional Cas9 systems [96,97]. Unlike DNA-targeting nucleases, Cas13 enzymes target RNA molecules and enable transient transcript regulation without permanent genome modification. In plants, Cas13 has been applied to confer resistance against RNA viruses, including *Tobacco mosaic virus* (TMV) and *Turnip mosaic virus* (TuMV), and has been used for transcript-level functional analyses in *Nicotiana benthamiana* and rice [98,99].

Another rapidly developing area is transcriptome and epitranscriptome engineering. While DNA editing remains the primary focus of crop improvement, Cas13-based systems have expanded CRISPR functionality to RNA molecules. Orthologs such as RfxCas13d (CasRx), Cas13x, and Cas13y enable efficient transcript knockdown and antiviral protection without permanent genome modification [100]. Furthermore, fusion of catalytically inactive Cas13 proteins with RNA methylation writers or erasers enables programmable m6A modification, providing a powerful platform for regulating gene expression and stress–response pathways [101].

Furthermore, base editing and prime editing technologies enable precise nucleotide substitutions, insertions, and deletions without generating double-strand breaks. Cytosine and adenine base editors have been successfully employed in rice, wheat, maize, tomato, and *Arabidopsis* to generate herbicide-resistance alleles and modify agronomically important traits through precise nucleotide substitutions [102,103,104]. Prime editing has subsequently been demonstrated in rice, wheat, tomato, and *Arabidopsis*, enabling targeted base substitutions as well as small insertions and deletions without requiring donor DNA templates [92,105,106,107,108]. Prime-editing technologies have likewise undergone substantial optimization. Nuclease-mediated prime editing (NM-PE) exploits microhomology-mediated end joining to facilitate precise sequence modifications, whereas advanced systems such as rPE20 and PEmax significantly improve editing efficiency through engineered reverse transcriptases and optimized Cas9 variants [109,110,111]. For larger genomic modifications, TwinPE and PrimeRoot enable insertion of DNA fragments exceeding 10 kb without generating double-strand breaks [110]. Editing outcomes remain influenced by environmental conditions, with certain Cas12a orthologs exhibiting enhanced activity at elevated temperatures [112].

Recent advancements in plant genome engineering have shifted the field from simple double-strand break induction toward highly precise and programmable genome manipulation [113,114,115,116,117]. Current developments focus on five major areas: engineering of novel CRISPR effectors, expansion of targeting scope, improvement of base and prime editing systems, enhancement of transformation and regeneration efficiency, and integration of genome editing with transcriptome engineering and computational design tools.

A major frontier is the development of compact and hypercompact CRISPR nucleases suitable for efficient delivery. The enCasδ nuclease, an evolutionary transitional system of approximately 900 amino acids, has been engineered to recognize a 5′-RYR-3′ PAM and achieves editing efficiencies of up to 80% in stable transgenic maize [118,119,120,121]. Similarly, the hypercompact en4Cas12j-8 (CasΦ) recognizes a 5′-TTN-3′ PAM and enables highly efficient base editing in soybean, reaching efficiencies above 90% without generating substantial indel by-products [122,123]. Additional compact systems, including CoCas9 and SpCas12f, have demonstrated robust editing performance in citrus and rice, respectively, highlighting their suitability for viral delivery applications [124,125,126,127].

In parallel, the targeting range of CRISPR systems has been broadened through the discovery and engineering of novel Cas variants. LrCas9, derived from Lactobacillus rhamnosus GG, recognizes a unique 5′-NGAAA-3′ PAM and exhibits high fidelity together with reduced temperature sensitivity [128]. In soybean, engineered SpCas9-NG and XNG-Cas9 variants further expand editing capabilities at non-canonical PAM sites [129]. Similarly, protein-engineered Cas12a variants such as LbCas12a-RRV efficiently recognize non-canonical TTV PAMs and can achieve near-complete editing efficiencies in rice and poplar [130].

Considerable progress has also been made in precision editing technologies. The Plant High-Efficiency Base Editor (PhieABE and PhieDBE) platforms employ evolved deaminases and DNA-binding domains to broaden editing windows and substantially increase editing efficiency [131,132]. The development of C-to-G base editors (CGBEs), particularly when combined with the near-PAMless SpRY variant, has enabled targeted transversion mutations at previously inaccessible genomic sites [133,134]. Further optimization of promoter architecture has improved editing efficiency even in complex polyploid genomes such as potato [135]. Base-editing precision has been additionally enhanced through phage-assisted evolution, resulting in highly purified editing systems with minimal by-product formation.

Beyond editing chemistry, transformation and regeneration remain major bottlenecks in many plant species. Recent advances employing morphogenic regulators, including GRF-GIF chimeras and REF1 peptide signaling systems, have markedly improved regeneration efficiency in previously recalcitrant crops such as wheat, maize, and citrus [136].

Finally, modern genome-engineering workflows increasingly integrate editing technologies with advanced selection, sequencing, and computational tools. Platforms such as pECNUS4 facilitate the rapid identification of transgene-free mutants, whereas nanopore Cas9-targeted sequencing (nCATS) enables high-resolution characterization of edited loci [134]. Machine-learning-based prediction tools, including CRISPRDB, RS3, and CRISPRon, further improve guide RNA design and support the emergence of AI-assisted, systems-level engineering strategies for the modification of complex polygenic traits [137].

An overview of different Cas9 variants applied in plant genome editing is summarized in Table 2.

## 4. Applications of Genome Editing in Plants

Collectively, these genome editing platforms enable functional characterization of genes, generation of precise mutant alleles, and targeted modification of endogenous genomic loci. In plant biotechnology, genome editing technologies provide powerful tools for improving agronomically important traits, including yield stability, resistance to biotic and abiotic stresses, nutrient-use efficiency, and product quality, thereby playing a central role in next-generation crop breeding strategies.

### Role of Genome Editing in Modern Crop Improvement

The integration of genome editing technologies into plant biotechnology represents a major advancement in crop improvement methodologies. Unlike conventional breeding, which is constrained by reproductive barriers, linkage drag, and long breeding cycles, genome editing enables precise and targeted modification of specific genomic regions. These approaches facilitate the rapid development of improved cultivars while preserving elite genetic backgrounds and minimizing unintended genetic changes [7]. In contrast, genetic engineering permits the direct insertion, deletion, or alteration of specific genetic loci, offering a time-efficient and species-independent platform for trait enhancement [96].

One of the most significant achievements of plant genetic engineering is the development of cultivars exhibiting improved agronomic traits, including enhanced yield stability, resistance to biotic and abiotic stresses, and improved adaptation to environmental conditions… Similarly, the incorporation of herbicide resistance genes, such as those conferring glyphosate or glufosinate tolerance, has streamlined weed management, facilitated reduced-tillage agriculture, and contributed to lower greenhouse gas emissions through minimized mechanical soil disturbance [99].

Genetic engineering has also played a pivotal role in addressing global nutritional deficiencies through metabolic pathway modifications and biofortification strategies. The development of Golden Rice, engineered to produce β-carotene in the endosperm, represents a landmark example in efforts to combat vitamin A deficiency in low-income populations [99,102]. Beyond provitamin A enhancement, transgenic approaches have also been applied to increase the content of essential micronutrients such as iron and zinc, as demonstrated in improved rice and wheat varieties [104]. Moreover, modifications aimed at increasing amino acid bioavailability (e.g., lysine- and methionine-enriched maize) underscore the versatility of genetic engineering in improving crop nutritional profiles [105].

Another critical area of impact lies in the reduction in post-harvest losses and extension of shelf life. Modulation of ripening pathways and suppression of endogenous enzymes involved in tissue softening—such as polygalacturonase—have yielded commercial varieties with enhanced storability, including the Flavr Savr tomato, which exhibited delayed softening and improved transport characteristics [106]. Such innovations contribute to reducing post-harvest losses throughout storage, transportation, and distribution systems.

Environmental sustainability may be further promoted through the implementation of genome editing technologies in crop improvement programs. In contrast to conventional transgenic approaches, genome editing enables precise modification of endogenous genetic loci, facilitating the development of cultivars with enhanced resistance to biotic and abiotic stresses, improved nutrient use efficiency, and reduced dependence on chemical crop protection measures. Such targeted genetic interventions have demonstrated considerable potential for improving agricultural productivity while minimizing environmental impacts associated with intensive crop production systems. Furthermore, the ability to introduce desirable traits without necessarily incorporating foreign DNA may facilitate the development of crop varieties that combine agronomic performance with environmental sustainability objectives [149,150].

In parallel, genetic engineering lays the groundwork for emerging fields such as plant synthetic biology and precision agriculture. Recent advances facilitate the rational design of synthetic gene circuits, targeted epigenetic regulation, and genome-scale reprogramming of metabolic networks. These developments enable the creation of ‘smart crops’ with dynamic responses to environmental stimuli, improved input efficiency, and tailored phenotypes for specific agroecosystems [151].

## 5. Botanical Description and Molecular Insights of *Linum usitatissimum* L

*Linum usitatissimum* L. (family *Linaceae*) is an annual, diploid (2n = 30) herbaceous species cultivated for both its bast fibers and oil-rich seeds. The plant exhibits an erect, glabrous stem ranging from 60 to 150 cm in height, with alternate, sessile leaves that are narrow, lanceolate, and entire-margined leaves that are narrow, lanceolate, and entire-margined [151,152,153,154]. The inflorescence is a terminal cyme bearing actinomorphic flowers with pentamerous symmetry. Floral organs include five free sepals and five blue to pale violet petals, ten stamens, and a superior ovary composed of five fused carpels [153].

Molecular studies utilizing SSR (simple sequence repeats) and SNP (single-nucleotide polymorphism) markers have revealed substantial genetic diversity within cultivated and wild flax germplasm, critical for breeding and genetic improvement programs. The species exhibits population structure reflecting both geographic origin and trait variation, facilitating the identification of loci associated with fiber quality and oil biosynthesis [155,156,157].

To provide a practical reference for flax genetic studies, Table 3 summarizes representative SSR and SNP molecular markers reported in *L. usitatissimum* L., including their sequences (where available), genomic distribution, and applications in genetic diversity analysis, linkage mapping, and trait association studies. These markers have been widely used for germplasm characterization, population structure analysis, and identification of loci associated with fiber quality and seed oil composition.

The integration of SSR- and SNP-based markers with genome sequencing data has significantly enhanced the resolution of flax genetic maps and accelerated the identification of trait-associated loci relevant for molecular breeding and genome editing strategies.

The flax genome was sequenced and assembled to a reference-quality standard, providing insight into gene families involved in lignin biosynthesis, fatty acid metabolism, and stress responses. This genomic resource supports genome editing and marker-assisted selection in breeding [161]. A high-quality, chromosome-scale genome assembly of the oil flax cultivar ‘Neiya No. 9’ was reported, generated using PacBio long-read sequencing, Hi-C data, and a genetic linkage map. The final assembly spans approximately 473.55 Mb, representing 94.7% of the estimated genome, and comprises 15 chromosomes with high contiguity (N50 of contigs: 0.91 Mb; N50 of scaffolds: 31.72 Mb) [162,163]. A total of 32,786 protein-coding genes were annotated, with 95.9% completeness based on BUSCO assessment, confirming the assembly’s reference-level quality. This genomic resource provided a platform for the first genome-wide association study (GWAS) of nuclear male sterility in flax. Phenotypic and cytological analyses revealed that male sterility in the studied population follows a dominant inheritance pattern and is associated with disrupted meiosis and premature tapetum degeneration. GWAS identified key candidate loci associated with this trait, including a cysteine synthase gene (*LUSG00017705*) with reduced expression in sterile lines, and a papain-like cysteine protease gene (*LUSG00017565*) harboring nonsynonymous mutations likely responsible for defective tapetal cell death and subsequent pollen abortion [92]. The integration of this genomic data with trait-mapping approaches represents a significant step toward functional gene discovery in flax. The identification of candidate genes underlying male sterility provides valuable targets for molecular breeding, including marker-assisted selection (MAS) and genome editing strategies. These findings have particular relevance for the development of hybrid flax varieties and for improving breeding efficiency through controlled fertility systems [163].

Flax is adapted to temperate climates, exhibiting a growth cycle of 90 to 120 days. The species demonstrates moderate drought tolerance, attributed in part to an extensive root system and stomatal regulation mechanisms that optimize water use efficiency [163,164,165]. Photosynthetically, flax operates as a C3 photosynthetic species, meaning that the initial stable product of CO_2_ fixation is the three-carbon compound 3-phosphoglycerate, formed via the Calvin–Benson cycle. This pathway is characterized by optimal enzymatic efficiency of RuBisCO under moderate environmental conditions. Consequently, flax exhibits maximal photosynthetic performance under moderate light intensities and temperatures ranging from 15 to 25 °C, where carbon assimilation is most efficient and photorespiratory losses remain relatively low. At higher temperatures, increased oxygenation activity of RuBisCO enhances photorespiration, thereby reducing net photosynthetic efficiency and limiting overall carbon gain [163,166]. Seed oil accumulation is tightly regulated through complex metabolic pathways involving fatty acid desaturases, notably *FAD3*, which catalyzes the formation of α-linolenic acid, a major component of flaxseed oil [167]. Studies in flax have identified two microsomal *FAD3* genes, *LuFAD3A* and *LuFAD3B*, which are directly responsible for converting linoleic acid (C18:2) to α-linolenic acid (C18:3) in seeds. These FAD3 desaturase enzymes play a central role in regulating linolenic acid levels, which typically range from ~45–65% of total seed fatty acids in traditional flax cultivars [167].

The availability of the flax reference genome and transcriptomic datasets has accelerated functional genomics studies, particularly using CRISPR/Cas9 technology. Emerging multiplex genome editing approaches facilitate simultaneous modifications of multiple loci, expediting trait pyramiding for complex characteristics.

## 6. Genome Editing in Flax: Applications and Achievements

### 6.1. Gene Delivery Systems for Genetic Transformation of Flax

The development of efficient genetic transformation systems in flax has become a central objective in modern plant biotechnology due to the increasing importance of this species as both an industrial fiber crop and a source of nutritionally valuable seed oil. Reliable gene delivery platforms are essential for functional genomics studies, metabolic engineering, and the implementation of genome editing technologies aimed at accelerating precision breeding strategies. Among the currently available methodologies, *Agrobacterium tumefaciens*-mediated transformation remains the most widely used and reliable approach for stable nuclear gene integration. *Agrobacterium tumefaciens*-mediated transformation remains the most widely used approach in flax, owing to its relatively high stability of T-DNA integration and broad applicability across explant types, including hypocotyls, cotyledons, leaf tissues, and immature embryos. In addition, *in planta* approaches such as floral-dip transformation have been developed as simplified alternatives to tissue culture-based methods [168,169].

Transformation efficiency in flax is strongly influenced by genotype, explant source, bacterial strain, and regeneration capacity. Hypocotyl-derived explants are generally regarded as the most responsive material due to their high morphogenic competence and regeneration potential. Several disarmed *Agrobacterium tumefaciens* strains (e.g., LBA4404, EHA105, GV3101) are used in flax, with transformation efficiency strongly influenced by strain virulence and host–bacterium compatibility. Differences in virulence gene composition and host compatibility significantly affect T-DNA delivery efficiency and stable integration [168,170,171].

In addition to *A*. *tumefaciens*, *A*. *rhizogenes* has been used to induce hairy root cultures in flax. This system is particularly useful for root-specific functional analyses and studies of secondary metabolism. However, its application for whole-plant regeneration and stable germline transmission remains limited because regenerated plants frequently exhibit altered morphology associated with Ri plasmid genes [172]. Alternative gene delivery systems have also been explored in flax. PEG-mediated transformation of protoplasts enables direct uptake of exogenous DNA into isolated cells and has been valuable for transient expression studies. Nevertheless, the practical applicability of protoplast-based transformation is restricted by low regeneration efficiency [100,173]. Particle bombardment (biolistic transformation) represents another genotype-independent delivery platform that enables direct DNA transfer into plant tissues. Despite its broad applicability, this method is often associated with multicopy insertions, genomic rearrangements, and unstable transgene expression [171].

Several methodological improvements have been introduced to enhance transformation efficiency in flax. Sonication-assisted *Agrobacterium*-mediated transformation (SAAT) improves bacterial penetration through ultrasound-induced micro-wounding of plant tissues, thereby increasing transformation frequency [170]. Furthermore, floral-dip and apical meristem transformation protocols have emerged as promising alternatives that bypass prolonged tissue culture and reduce somaclonal variation [169].

Collectively, flax transformation systems encompass biological, chemical, and physical gene delivery platforms. Although *Agrobacterium*-mediated transformation remains the gold standard for stable genetic engineering in flax, ongoing optimization of delivery systems and tissue culture protocols continues to expand the experimental toolbox available for functional genomics and molecular breeding.

### 6.2. In Vitro Regeneration and Tissue Culture Systems in Flax for Genetic Transformation

Efficient in vitro regeneration systems constitute a fundamental prerequisite for successful genetic transformation and genome editing in flax. The regeneration capacity of transformed tissues directly determines the feasibility of stable transgene integration and recovery of edited plants. However, flax is considered a moderately recalcitrant species under tissue culture conditions because morphogenic responses are strongly dependent on genotype, explant source, physiological state of donor tissue, and culture conditions [103,104,105].

Early studies demonstrated that whole flax plants could be regenerated from a broad range of explants, including hypocotyls, cotyledons, immature embryos, anthers, and protoplast-derived callus tissues. Among these systems, hypocotyl explants later became the preferred material for transformation due to their high regenerative capacity and reproducibility [172,174].

Regeneration efficiency in flax is strongly influenced by the composition of plant growth regulators in the culture medium. Cytokinins such as thidiazuron (TDZ) and 6-benzylaminopurine (BAP), together with auxins including α-naphthaleneacetic acid (NAA) and 2,4-dichlorophenoxyacetic acid (2,4-D), play critical roles in controlling organogenesis and somatic embryogenesis. Low concentrations of TDZ generally promote shoot regeneration, whereas elevated auxin levels stimulate callus proliferation and embryo-like structure formation. However, many embryo-like structures exhibit abnormal morphology and low conversion rates into viable plants [174].

A significant advancement in flax biotechnology was achieved through the development of *Agrobacterium*-mediated transformation systems. Basiran et al. (1987) [168] successfully established genetic transformation of flax using *Agrobacterium tumefaciens* harboring a disarmed Ti plasmid vector. In their study, transformed shoots were regenerated indirectly through a callus-mediated organogenic pathway initiated from hypocotyl explants. Molecular analyses, including Southern blot hybridization, confirmed stable integration of T-DNA into the flax genome, while expression of selectable marker genes such as nptII verified the transgenic status of regenerated plants. This work represented one of the earliest reproducible transformation systems developed for flax and laid the foundation for subsequent genetic engineering studies [168].

Despite significant methodological progress, several biological limitations continue to constrain flax regeneration efficiency. One of the major obstacles is strong genotype dependency, whereby individual cultivars respond differently under identical culture conditions. Prolonged callus proliferation frequently leads to somaclonal variation, chromosomal instability, epigenetic alterations, and abnormal plant development [175].

Oxidative stress also represents an important factor affecting regeneration success. Mechanical injury during explant excision and bacterial infection induce the accumulation of reactive oxygen species (ROS), which frequently causes tissue browning, cellular damage, and reduced morphogenic competence. Antioxidative compounds such as desferrioxamine have been shown to partially alleviate oxidative stress and improve regeneration efficiency under specific experimental conditions [176].

In addition to oxidative stress, regeneration failure is associated with cellular disorganization and physiological instability. Disturbances in endoplasmic reticulum integrity and stress–response pathways can impair protein processing and reduce the stability of reporter gene expression during regeneration.

Selection pressure applied during transformation further influences regeneration outcomes. Although antibiotic or herbicide selection is necessary for identifying transformed cells, excessive selection pressure may inhibit regeneration or promote the formation of chimeric tissues.

To overcome limitations associated with tissue culture-induced variation, alternative transformation systems independent of extensive callus phases have recently attracted considerable attention. Floral-dip and apical meristem-targeted transformation strategies reduce prolonged dedifferentiation and minimize somaclonal variation while maintaining stable transgene integration.

Recent studies increasingly focus on optimizing endogenous hormonal balance, antioxidative capacity, and culture conditions to improve regeneration efficiency. Such improvements are expected to enhance the reproducibility of transformation systems and facilitate the application of genome editing technologies in flax [177,178]. To provide a structured overview of regeneration systems used in flax, Table 4 summarizes previously reported protocols based on explant type, culture media, hormonal composition, and regeneration efficiency. This compilation highlights the strong influence of genotype and culture conditions on regeneration outcomes, which represent major limiting factors for the application of genome editing technologies in flax. In particular, protoplast-based systems remain inefficient and technically challenging, restricting their use for stable CRISPR/Cas-mediated plant regeneration.

Overall, the data summarized in Table 4 indicate that regeneration efficiency in flax remains a critical bottleneck for genome editing applications, particularly in approaches requiring callus formation or protoplast regeneration.

### 6.3. Selectable and Reporter Genes Used in Flax Transformation

Selectable and reporter genes represent indispensable components of flax transformation systems because they facilitate the identification of transformed cells and enable monitoring of transgene expression throughout regeneration and molecular characterization procedures.

The most widely used selectable marker in flax is the neomycin phosphotransferase II (nptII) gene, which confers resistance to aminoglycoside antibiotics such as kanamycin. This system has been extensively employed in both early and contemporary flax transformation studies and remains one of the most reliable selection approaches [182].

Concerns regarding biosafety and environmental impacts associated with antibiotic resistance markers have stimulated the development of alternative selection systems. One of the most important alternatives is the phosphomannose isomerase (*pmi*)/mannose system, in which transformed cells metabolize mannose as a carbon source and survive on selective media. This approach provides selection efficiency comparable to antibiotic-based methods while avoiding the use of antibiotic resistance genes [183].

Reporter genes are widely used for visualization and molecular analysis of transgene expression. The β-glucuronidase (*GUS*, *uidA*) reporter system remains one of the most commonly used markers in flax transformation studies because it enables simple histochemical detection of gene expression patterns [182].

Green fluorescent protein (GFP) has also been employed for non-destructive visualization of transgene expression in living tissues. However, stable GFP expression in flax can be difficult to maintain during prolonged regeneration and callus development, indicating that reporter stability is influenced by physiological stress and tissue culture conditions [184].

Collectively, selectable and reporter systems constitute indispensable components of flax transformation and genome editing workflows, facilitating molecular validation, optimization of transformation protocols, and evaluation of CRISPR/Cas-mediated editing efficiency.

### 6.4. Promoter Resources and Their Application in CRISPR/Cas-Mediated Genome Editing of Flax

Efficient implementation of CRISPR/Cas-based genome editing systems in flax depends critically on the availability of promoters capable of ensuring stable and sufficient expression of both Cas nucleases and guide RNAs. Promoter selection is therefore a key determinant of editing efficiency, mutational stability, and reproducibility of genome engineering workflows. Compared with model plant species, the repertoire of fully characterized flax-specific promoters remains limited, and many transformation systems continue to rely on heterologous regulatory elements [185].

The Cauliflower mosaic virus 35S (CaMV 35S) promoter is the most widely used constitutive promoter in flax genetic engineering. Due to its strong transcriptional activity across multiple tissues, it has been extensively applied for the expression of selectable marker genes and Cas nucleases. Nevertheless, because CaMV 35S is of viral origin, concerns remain regarding transcriptional variability and transgene silencing during long-term stable expression [186].

Constitutive ubiquitin (UBQ) and actin (ACT) promoters derived from heterologous species have also been employed in flax functional genomics studies. These promoters generally provide relatively stable expression in vegetative tissues, although their efficiency may vary depending on genomic context and transformation strategy.

A major advancement in flax genome editing has been the identification and functional characterization of endogenous U6 small nuclear RNA promoters. These promoters are essential for RNA polymerase III-driven expression of single-guide RNAs (sgRNAs) in CRISPR/Cas systems. Comparative analyses revealed multiple U6 loci in the flax genome, with substantial variation in promoter activity among family members [185].

Functional studies demonstrated that flax-derived U6 promoters outperform heterologous promoters such as *Arabidopsis thaliana* U6 in flax transformation systems. Moreover, truncated promoter variants approximately 300–350 bp in length retained high transcriptional activity while reducing vector size, thereby improving construct design for multiplex genome editing [185].

Comparative genomic analyses indicate that flax promoters, including U6 and protein-coding gene promoters, contain conserved regulatory motifs such as upstream sequence elements (USE), TATA boxes, and CAAT-box motifs. These cis-regulatory elements are critical for transcription initiation and efficiency, and their arrangement is highly conserved relative to model plant species such as *Arabidopsis thaliana* [20]. In addition to U6 promoters, flax genome studies have identified regulatory regions associated with genes involved in stress responses and secondary metabolism. These promoters are of particular interest for engineering traits such as fiber quality, lignan biosynthesis, and abiotic stress tolerance, although most remain functionally uncharacterized at the level required for routine genome editing applications [187,188].

Promoter selection represents one of the key determinants of genome editing efficiency in flax. Typically, Cas9 expression is controlled by constitutive promoters such as CaMV 35S or UBQ promoters, whereas sgRNA transcription depends on U6 or U3 RNA polymerase III promoters. The use of endogenous flax U6 promoters substantially improves sgRNA abundance and editing efficiency in transformed tissues [185].

Although flax still lacks a comprehensive library of tissue-specific and inducible promoters, recent characterization of endogenous regulatory elements represents a major step toward improving CRISPR/Cas applications in this species. Continued promoter discovery and functional validation are expected to enhance editing precision and facilitate advanced genome engineering strategies [185,189,190].

### 6.5. Non-CRISPR RNAi and TILLING Approaches for Functional Gene Analysis in Flax

Prior to the widespread implementation of CRISPR/Cas technologies, functional characterization of genes in flax was predominantly conducted using RNA interference (RNAi) and TILLING-based approaches. These methodologies established the foundation for reverse genetics studies and contributed substantially to the identification of genes associated with fiber development, lignin biosynthesis, and stress responses. These methodologies provided important insights into genes associated with lignin biosynthesis, fiber development, and stress responses [191].

RNAi-mediated suppression of cinnamyl alcohol dehydrogenase (*CAD*) resulted in substantial reductions in lignin content, accompanied by modifications in secondary cell wall architecture. These alterations included increased cellulose and pectin deposition, improved tensile strength, and more uniform retting processes. However, reduced lignification was also associated with slightly increased susceptibility to *Fusarium* infection [192].

Similar studies targeting caffeic acid O-methyltransferase (*COMT*) and *CAD* homologs in other plant species demonstrated altered lignin composition and modified syringyl-to-guaiacyl ratios, thereby improving enzymatic digestibility and biomass processing [193].

Unlike CRISPR/Cas-mediated mutagenesis, RNAi generally induces partial and variable suppression of gene expression rather than complete gene knockout. Consequently, phenotypic outcomes are often less stable and more difficult to reproduce across experimental systems.

TILLING approaches based on chemically induced mutagenesis have also contributed to flax functional genomics by enabling the identification of allelic variants and mutant populations. Although these strategies remain valuable for reverse genetics studies, they are considerably more time-consuming and less precise than contemporary genome editing technologies.

### 6.6. Established CRISPR/Cas9 Genome Editing Platforms and Targeted Gene Validation in Flax

The introduction of CRISPR/Cas9 technology has established the methodological foundation for precise genome engineering in flax. In comparison with earlier transformation and gene-silencing approaches, CRISPR/Cas systems enable targeted and heritable modifications of endogenous loci with substantially higher precision and reproducibility. Experimentally validated genome editing studies in this species currently focus primarily on proof-of-concept modifications and optimization of transformation components.

One of the first successful CRISPR/Cas9 genome editing platforms in flax targeted the phytoene desaturase genes *LuPDS1* and *LuPDS2*. These genes were selected as visual marker loci because disruption of phytoene desaturase activity results in an albino phenotype that can be easily identified. Specific sgRNAs targeting endogenous loci were introduced using *Agrobacterium tumefaciens*-mediated transformation [22].

Molecular analyses in reported studies indicate successful targeted mutagenesis, although editing efficiency remains highly genotype-dependent. Sequencing analyses verified stable editing events, while phenotypic characterization further confirmed the functional consequences of induced mutations. This work established a reliable methodological framework for genome editing and demonstrated that heritable CRISPR/Cas9-mediated mutagenesis is feasible in flax.

To date, published CRISPR/Cas9 editing studies in flax have focused primarily on confirming on-target mutagenesis through PCR-based sequencing and phenotypic characterization. Systematic genome-wide off-target analysis—such as whole-genome sequencing of edited lines or unbiased detection methods including GUIDE-seq or CIRCLE-seq—has not yet been reported in flax. This represents a notable gap in the current literature, particularly given that off-target effects may confound phenotypic interpretation in a species where transformation and regeneration are already technically demanding. Future studies should incorporate rigorous off-target profiling as a standard component of flax genome editing workflows, especially for targets intended for applied breeding programs.

It should be noted that while CRISPR/Cas9-mediated genome editing has been successfully demonstrated in flax for selected proof-of-concept targets, the number of fully characterized, trait-stable edited lines remains limited. In particular, several reported gene targets, including those associated with herbicide resistance and metabolic pathway modification, are currently supported by preliminary or early-stage experimental evidence. Therefore, these findings should be interpreted as emerging applications rather than fully validated agronomic traits in flax [2,114].

Further improvements in editing efficiency were achieved through optimization of endogenous flax U6 promoters controlling sgRNA expression. Truncated flax-derived U6 promoter variants significantly enhanced guide RNA transcription compared with commonly used heterologous promoters, thereby increasing overall editing efficiency and consistency.

Collectively, these studies demonstrate that stable and efficient CRISPR/Cas9-mediated genome editing can be achieved in flax and provide a foundational platform for future functional genomics and molecular breeding applications.

### 6.7. Prospects of CRISPR-Based Genome Editing for Flax Breeding Improvement

**Figure 2:**
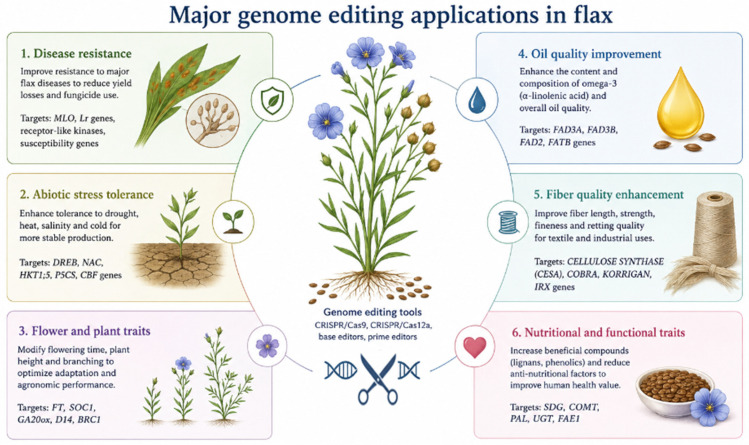
Major genome editing applications in flax and candidate target genes for trait improvement. The six priority areas include disease resistance, abiotic stress tolerance, flower and plant traits, oil quality improvement, fiber quality enhancement, and nutritional and functional traits. Genome editing tools applicable to these targets include CRISPR/Cas9, CRISPR/Cas12a, base editors, and prime editors.

CRISPR/Cas-mediated genome editing represents one of the most promising strategies for the future improvement of agronomic and industrial traits in flax. The ability to introduce targeted and heritable modifications into endogenous genes provides unprecedented opportunities for accelerating breeding programs and dissecting complex biological pathways associated with fiber development, oil biosynthesis, and environmental adaptation. One of the most promising applications involves targeted modification of genes associated with fatty acid metabolism, particularly fatty acid desaturase genes such as *FAD3*, which regulate α-linolenic acid biosynthesis and determine seed oil composition [167]. The major genome editing applications in flax, together with the potential target genes representing priority areas for flax breeding improvement, are graphically presented in Figure 2.

Genome editing also provides opportunities to improve fiber-related characteristics, including tensile strength, fineness, retting efficiency, and lignin composition. Candidate targets include genes involved in lignin biosynthesis, particularly *CAD* and *COMT*, which have previously been investigated through RNAi-based functional studies [192].

Another promising area involves engineering genes associated with cellulose biosynthesis and cytoskeletal organization. The cellulose synthase (CesA) and tubulin gene families play central roles in cellulose microfibril deposition and secondary cell wall formation. However, extensive genetic redundancy within these multigene families complicates conventional functional analyses [194,195,196].

Advanced genome editing approaches such as multiplex CRISPR/Cas systems enable simultaneous targeting of multiple homologous genes, thereby overcoming limitations associated with gene redundancy. Base editing and prime editing technologies further expand the precision of genome engineering by allowing targeted nucleotide substitutions without generating double-strand breaks [197,198].

Genome editing may also facilitate the development of flax cultivars with enhanced tolerance to abiotic stresses such as drought, salinity, and temperature fluctuations. These traits are becoming increasingly important in the context of climate change and sustainable agriculture [199,200].

The integration of optimized regeneration systems, endogenous promoter engineering, multiplex editing, and transgene-free genome editing approaches is expected to substantially accelerate flax improvement. Continued development of CRISPR/Cas technologies will provide powerful tools for functional genomics and precision breeding in this economically important crop [6,185,201].

Furthermore, the implementation of multiplex genome editing allows simultaneous targeting of multiple homologous genes, while base editing enables subtle, non-disruptive nucleotide changes that preserve gene expression [202]. The current state of genome editing systems established in flax is comprehensively summarized in Table 5.

In addition, genome editing targets such as *EPSPS* and *ALS*, which are well-established in other crop species for herbicide resistance engineering, have been proposed for flax improvement. However, in flax, these applications remain largely at the experimental or proof-of-concept stage, and stable field-validated phenotypes have not yet been comprehensively demonstrated. Consequently, these targets should be considered as translational rather than fully validated applications in flax [203].

**Table 5:** Genome editing in flax (*Linum usitatissimum* L.): validated applications and transferable approaches.

**A. Experimentally Validated in Flax**									
**System**	**Target Gene(s)**	**Trait/Phenotype**	**Delivery Method**	**Promoter(s)**	**Editing Outcome**	**Validation Method**	**Key Limitations**	**Functional Interpretation**	**References**
CRISPR/Cas9	*LuPDS1, LuPDS2*	Albino knockout phenotype	*Agrobacterium tumefaciens*	CaMV 35S/U6	Efficient targeted mutagenesis in T0 plants	PCR, sequencing, phenotype	Regeneration bottleneck; genotype dependency	Standard proof-of-concept system for flax genome editing	[22]
CRISPR/Cas9	*LuALS*	Herbicide (sulfonylurea) resistance	*Agrobacterium tumefaciens*	U6	Targeted mutations conferring tolerance	Sequencing, herbicide assay	Transformation variability	Amino acid biosynthesis pathway engineering	[204]
CRISPR/Cas9 (optimized)	Endogenous U6 promoters	Increased sgRNA expression	*Agrobacterium tumefaciens*	Flax-specific U6	Improved editing efficiency vs. heterologous promoters	Mutation frequency analysis	Limited promoter characterization	Key enabling tool for flax genome editing	[185]
RNAi	*CAD*	Reduced lignin content; fiber modification	*Agrobacterium tumefaciens*	35S	Strong gene silencing phenotype	Lignin assays, microscopy	Off-target physiology changes	Functional validation of lignin biosynthesis pathway	[192]
TILLING + CRISPR validation	Multiple loci	Reverse genetics platform	Chemical mutagenesis + CRISPR	N/A	Mutant identification and confirmation	Genotyping, phenotyping	Time-consuming population development	Functional genomics resource in flax	[191]
**B. Translatable Approaches (Validated in Other Plants, Not Yet Fully Established in Flax)**									
**System**	**Target Gene(s)**	**Trait/Phenotype**	**Delivery Method**	**Promoter(s)**	**Expected Outcome in Flax**	**Evidence Base**	**Key Limitations in Flax**	**Functional Interpretation**	**References**
CRISPR/Cas9	*EPSPS*	Glyphosate tolerance	*Agrobacterium*	U6	Targeted herbicide resistance alleles	Validated in multiple crops (rice, maize, soybean)	Limited flax-specific functional validation	Metabolic engineering target requiring flax confirmation	[22,142,205]
CRISPR/Cas9	*FAD3A/FAD3B*	Altered α-linolenic acid content	*Agrobacterium*/transient	Endogenous/heterologous	Modified oil composition expected	Functional validation in model crops + partial flax evidence	Functional redundancy in flax genome	Lipid biosynthesis engineering target	[206,207]
CRISPR/Cas9/RNAi	*COMT*	Altered lignin composition	*Agrobacterium*	Constitutive promoters	Modified lignin S/G ratio expected	Validated in *Arabidopsis* and cereals	Pleiotropic effects likely in flax	Monolignol pathway engineering	[208,209]
Multiplex CRISPR/Cas9	Tubulin gene family	Fiber/cytoskeleton modification	Protoplast/*Agrobacterium*	U6 multiplex cassettes	Complex trait modification expected	Demonstrated in model plants	High redundancy limits efficiency in flax	Structural gene family editing strategy	[210]
Multiplex CRISPR/Cas9	*CesA* gene family	Cellulose biosynthesis modulation	*Agrobacterium*/RNP	U6 promoters	Cell wall engineering expected	*Arabidopsis* and biomass crops	Strong redundancy; regeneration limits	Cellulose pathway engineering	[211]

Validated in flax—refers to experimentally confirmed gene editing or functional suppression in stable flax systems. Translatable approaches—refer to targets and genome editing strategies validated in other plant species with strong mechanistic relevance but limited or preliminary experimental validation in flax.

## 7. Discussion

The findings reviewed in this work collectively illustrate that flax is transitioning from a species with limited genomic resources to one with an increasingly robust genome editing infrastructure. The successful targeting of *LuPDS1*/*LuPDS2* and *LuALS* confirms that heritable CRISPR/Cas9-mediated mutagenesis is feasible in flax; however, these achievements represent a narrow subset of the candidate gene space with genuine agronomic relevance. High-value targets, such as *FAD3A*/*FAD3B* for oil composition, *CAD* and *COMT* for fiber lignification, and members of the *CesA* and *tubulin* gene families for secondary cell wall architecture, currently lack stable edited flax lines with confirmed agronomic phenotypes. The primary challenge for the field has therefore shifted from system establishment toward systematic functional validation of these targets across multiple genetic backgrounds.

Achieving this goal depends critically on overcoming the low efficiency of homology-directed repair (HDR) in plant cells. Although CRISPR/Cas systems provide highly programmable DNA cleavage, repair outcomes in plant somatic cells are predominantly governed by the error-prone non-homologous end joining (NHEJ) pathway, which severely restricts the frequency of precise genetic modifications. In flax, this limitation is further compounded by inherently low transformation efficiency and pronounced genotype dependence, making the recovery of precisely edited lines particularly challenging. Several strategies have been shown to improve HDR frequency in other plant systems and warrant systematic evaluation in flax. These include geminiviral replicon-based donor amplification to increase template copy number, cell-cycle synchronization toward S/G2 phases where homologous recombination activity is naturally elevated, and partial suppression of core NHEJ factors—KU70, KU80, and DNA ligase IV (Lig4)—to bias repair outcomes toward HDR-mediated pathways. Beyond pathway modulation, donor template architecture itself significantly influences recombination frequency; parameters such as homology arm length, arm symmetry, strand configuration (single- versus double-stranded), and overall template topology have all been demonstrated to affect editing outcomes and require optimization within flax-specific systems [28,212,213].

In parallel, paired CRISPR/Cas9 nickase systems offer a complementary approach to increasing editing precision while minimizing off-target activity. Cas9 nickases, generated by inactivation of either the HNH or RuvC nuclease domain, introduce single-strand breaks rather than double-strand breaks. When two appropriately spaced guide RNAs are employed, paired nickases generate staggered DNA lesions that promote HDR while substantially reducing the risk of unintended genomic alterations—a strategy successfully applied in Arabidopsis, rice, and other plant species [214,215]. Similarly, single-stranded oligodeoxynucleotide (ssODN) donors have emerged as efficient repair templates for introducing precise nucleotide substitutions and small sequence modifications. Compared with conventional double-stranded DNA donors, ssODNs are easier to deliver, reduce the risk of random integration events, and can improve the frequency of targeted sequence replacement under specific experimental conditions, as demonstrated in rice and *Arabidopsis*. Although neither paired nickase systems nor ssODN-mediated repair have been extensively evaluated in flax, their successful application in model species provides a strong rationale for their adaptation to flax genome engineering platforms [216,217].

Importantly, base editing and prime editing circumvent HDR constraints entirely by introducing precise nucleotide changes without generating double-strand breaks or requiring donor templates [218,219]. Their deployment in flax could substantially accelerate trait engineering once species-specific delivery systems are further optimized, and they represent perhaps the most immediately tractable route to precise allele engineering in a species where HDR efficiency remains low.

Rapid advances in plant genome-editing technologies further strengthen this perspective. Recent developments have focused on expanding editing precision, target accessibility, and delivery efficiency through the engineering of compact nucleases, advanced base editors, and next-generation prime-editing systems. Hypercompact effectors such as Cas12f, CasΦ, enCasδ, and CoCas9 are particularly attractive because their reduced size facilitates viral-vector delivery and transgene-free genome engineering. Simultaneously, high-efficiency base-editing platforms, including PhieABE/PhieDBE and C-to-G base editors, now enable precise nucleotide substitutions at previously inaccessible genomic loci, while improved prime-editing systems support targeted sequence insertion without relying on HDR. Importantly, genotype-dependent regeneration—one of the major bottlenecks identified in flax—can increasingly be mitigated through morphogenic regulators such as GRF-GIF chimeras and REF1-mediated cellular reprogramming. Additional innovations, including CRISPR-Cas13-based transcriptome engineering, visible marker-assisted recovery of transgene-free plants, multiplex editing platforms, and machine-learning-guided gRNA design, are transforming genome editing into a comprehensive and highly programmable breeding technology. Although most of these systems have not yet been systematically evaluated in flax, their successful implementation in cereals, legumes, woody species, and fiber crops provides a strong foundation for future adaptation and suggests that many of the current limitations of flax genome engineering may be overcome through the integration of these next-generation editing platforms.

Of all biological constraints, however, cultivar-specific variation in regeneration competence is arguably the most practically limiting factor. Even high molecular editing efficiency is of little value if edited cells cannot be regenerated into fertile plants. Two complementary strategies merit prioritization: systematic screening of agronomically relevant cultivars under optimized phytohormonal conditions, and development of genotype-independent delivery approaches—such as ribonucleoprotein (RNP)-mediated transformation or meristem targeting—which reduce dependence on prolonged callus phases and minimize the risk of somaclonal variation [220].

Recent advances in plant genome-editing delivery technologies further support the feasibility of overcoming these bottlenecks. A major trend is the development of tissue culture-independent transformation systems, including de novo meristem induction through developmental regulators such as BBM, WUS2, and GRF4/GIF1, as well as genotype-independent transformation platforms that reduce reliance on labor-intensive regeneration procedures. Closely related approaches based on in planta genome editing enable direct delivery of CRISPR reagents to meristematic tissues, floral organs, pollen, or developing embryos, thereby bypassing callus formation and reducing genotype dependence. Additional innovations, including virus-induced genome editing (VIGE), nanocarrier-mediated delivery, compact genome editors compatible with viral vectors, and DNA-free ribonucleoprotein (RNP) systems, offer promising routes toward efficient transgene-free editing. Collectively, these technologies address several of the key limitations currently restricting flax genome engineering—namely, low transformation efficiency, genotype specificity, and regeneration constraints—and may substantially accelerate the development of precisely edited flax cultivars once adapted and validated in flax-specific transformation platforms.

To contextualize the current state of flax genome editing, it is instructive to compare progress in related fiber and oilseed crops. In cotton (*Gossypium hirsutum*), CRISPR/Cas9 has been successfully applied to improve fiber quality, enhance disease resistance, and modify fatty acid profiles, benefiting from well-established transformation protocols and extensive genomic resources [221]. Camelina (*Camelina sativa*) has emerged as one of the most genome-editing-amenable species among non-model plants, with multiple validated studies demonstrating precise modification of fatty acid desaturase genes and substantial shifts in oil composition achieved within relatively short experimental timelines [222]. Hemp (*Cannabis sativa*), despite its recent regulatory liberalization and growing industrial interest, remains at an early stage of genome editing development, constrained by limited transformation protocols and fragmented genomic resources [223]. Relative to these species, flax occupies an intermediate position: it benefits from a sequenced reference genome and established transformation systems, but lags behind camelina and cotton in the number of fully validated edited lines. This comparison underscores both the realistic near-term potential of flax genome editing and the specific bottlenecks—regeneration efficiency and genotype dependence—that must be resolved to reach parity with more advanced species.

Cross-species extrapolation of editing outcomes must nonetheless be approached with appropriate caution. Mechanistic rationale derived from work in *Arabidopsis* thaliana, rice, or soybean constitutes a valuable hypothesis-generating framework, but flax-specific factors—including functional redundancy within gene families, distinct secondary metabolite profiles, and regeneration constraints—may substantially alter phenotypic outcomes [5,114,117]. For example, *FAD3* knockout phenotypes well documented in soybean may be attenuated in flax due to compensatory activity between *LuFAD3A* and *LuFAD3B* paralogs [167]. Multiplex editing of *CesA* or *tubulin* gene families, effective in Arabidopsis, may similarly require higher-order simultaneous edits in flax to produce discernible cell wall phenotypes, given the greater redundancy within these families [224].

These considerations do not diminish the translational value of model-species findings but underscore the necessity of flax-specific experimental validation before agronomic claims can be advanced. Within this broader context of genome engineering strategies available for flax improvement, it is also important to consider the relative strengths and limitations of earlier programmable nuclease platforms.

Zinc-finger nucleases represent one of the earliest programmable nuclease platforms; however, their complex protein engineering requirements and limited scalability significantly restricted their widespread adoption in plant genome editing. TALENs, while technically more demanding than CRISPR/Cas systems, retain a practical advantage in targeting AT-rich or PAM-constrained genomic regions, as the absence of protospacer adjacent motif requirements expands the accessible target space. Their demonstrated efficacy in editing lipid metabolism genes such as *FAD2* in camelina and rapeseed provides a mechanistically relevant precedent for analogous applications in flax. Despite being largely superseded by CRISPR/Cas systems, TALENs therefore remain relevant for applications requiring high targeting flexibility and PAM-independent genome access [225,226].

It is equally important to recognize that CRISPR/Cas systems do not render pre-existing functional genomics tools obsolete—rather, they complement them. RNAi remains valuable for partial, tunable suppression of genes where complete knockout would be pleiotropically deleterious, as illustrated by *CAD* silencing in flax, which improved fiber properties while simultaneously increasing susceptibility to *Fusarium* infection. Analogous RNAi-mediated regulation of secondary cell wall biosynthetic pathways in cotton (*Gossypium hirsutum*) further highlights the broader utility of this approach in fiber crop engineering [192,227,228].

Collectively, genome engineering technologies have evolved from ZFNs to TALENs and ultimately to CRISPR/Cas systems, driven largely by increasing ease of design, efficiency, and multiplexing capability. Nevertheless, complementary approaches such as RNAi continue to provide valuable opportunities for tunable gene suppression when complete loss-of-function mutations are undesirable. The combined use of these tools therefore provides a versatile framework for dissecting gene function and engineering complex traits in flax, particularly within interconnected pathways such as monolignol and fatty acid metabolism. The regulatory environment will also strongly shape the commercial trajectory of genome-edited flax. Regulatory frameworks governing genome-edited plants vary considerably among jurisdictions. In the European Union, genome-edited organisms are currently regulated under Directive 2001/18/EC following the 2018 ruling of the Court of Justice of the European Union (Case C-528/16), although the recently proposed New Genomic Techniques (NGT) regulation may introduce differentiated requirements for certain categories of edited plants [229,230]. Similarly, in New Zealand, genome-edited organisms are regulated under the Hazardous Substances and New Organisms Act 1996, with requirements broadly comparable to those applied to transgenic organisms. In contrast, several countries have established regulatory exemptions for transgene-free genome-edited plants. In the United States, the USDA SECURE Rule (2020) exempts from GMO oversight plants carrying genetic changes that could otherwise be achieved through conventional breeding, thereby facilitating their commercialization. Argentina was among the first countries to implement a case-by-case regulatory determination process that excludes transgene-free edited plants from GMO regulations [231]. Likewise, Japan, Australia, and Brazil have adopted regulatory frameworks that distinguish genome-edited plants lacking foreign DNA from conventional transgenic organisms [232]. These regulatory disparities create uncertainty for the international trade and commercialization of genome-edited crops, including flax.

For flax, this regulatory divergence is of particular practical significance. Genome-editing approaches such as ribonucleoprotein (RNP)-mediated delivery, base editing, and prime editing can generate transgene-free plants, potentially positioning edited flax varieties more favorably within emerging regulatory pathways. Consequently, proactive alignment of experimental design with anticipated regulatory developments—particularly through the adoption of DNA-free editing strategies whenever technically feasible—may serve both scientific and commercial objectives. Furthermore, the development of harmonized international regulatory frameworks based primarily on product characteristics and trait outcomes rather than the breeding process itself would facilitate the responsible deployment and global commercialization of genome-edited flax varieties.

Beyond regulatory frameworks, the intellectual property (IP) landscape surrounding CRISPR/Cas technologies presents an additional consideration for flax genome editing research and commercialization. Key CRISPR/Cas9 patents are held by competing academic and commercial entities, and licensing arrangements differ substantially between jurisdictions and intended applications. For public breeding programs and small-to-medium enterprises focused on flax improvement, access to foundational CRISPR IP may represent a practical constraint, particularly in markets where patent enforcement is stringent. The emergence of alternative genome editing platforms—including base editors, prime editors, and Cas12a-based systems covered by distinct patent portfolios—may offer more accessible licensing pathways for certain applications. Proactive engagement with IP considerations during experimental design is therefore advisable for research groups aiming to translate flax genome editing outcomes into commercial cultivar development [2].

Looking forward, the integration of endogenous U6 promoter optimization [85], multiplex editing capability, improved regeneration protocols, and transgene-free delivery systems provides a realistic pathway toward functional validation of priority targets in the near term. The most tractable near-term objectives are oil composition engineering via *FAD3* editing and herbicide tolerance via *ALS* modification, both of which benefit from strong mechanistic precedents and defined selection strategies. Longer-term goals—including multiplex fiber quality improvement and abiotic stress tolerance engineering—will require deeper functional genomics work and the development of flax-specific resources such as tissue-specific promoter libraries and improved reference genomes spanning diverse cultivars. Collectively, these advances are expected to position flax as a fully genome-editing-amenable species and to accelerate its development as a sustainable dual-purpose crop for fiber and nutritional oil production [52,233].

## 8. Conclusions

Overall, although flax genome engineering is still less advanced compared to major model and crop species, rapid progress in genome editing tools, DNA repair pathway manipulation, and transformation technologies is steadily expanding its potential. Continued interdisciplinary efforts integrating plant molecular biology, biotechnology, and genome engineering will be essential to overcome current technical barriers and fully enable precise genetic improvement of flax.

Beyond technical optimization, the long-term success of flax improvement also depends on targeted enhancement of agronomic and quality traits. Key priorities include improved biomass yield, enhanced tolerance to abiotic stresses such as drought and salinity, and increased nutrient use efficiency to ensure stable productivity under variable environmental conditions.

In addition, fiber quality remains a central breeding objective, particularly regarding fiber length, strength, and uniformity. Improvements in these traits would significantly enhance industrial processing efficiency and textile performance. Similarly, seed-related traits such as oil content, fatty acid composition, and oxidative stability are critical for expanding flax applications in nutritional and functional food markets.

Disease and pest resistance also represent important targets for future improvement, as they directly affect yield stability and reduce dependence on chemical crop protection. Compared with intensively bred cereal crops, flax remains relatively vulnerable to biotic stresses, highlighting the need for more systematic genetic improvement in this area.

Realistic estimation of timelines for commercial deployment of genome-edited flax varieties must account for the multiple sequential steps required between initial editing and market release. Under optimistic but technically grounded assumptions—assuming continued optimization of transformation and regeneration protocols within the next two to three years—the first trait-validated edited flax lines for well-defined single-gene targets such as *ALS*-based herbicide tolerance or *FAD3*-modified oil composition could realistically be available for advanced field evaluation within five years. Regulatory review and variety registration, depending on jurisdiction and applicable legislation, would add a further two to five years before commercial release. More complex multigenic traits such as multiplex fiber quality improvement or combined abiotic stress tolerance are likely to require a longer development horizon of ten or more years, reflecting both the greater technical demands of multiplex editing and the more complex phenotypic validation required. These projections highlight the importance of initiating parallel tracks of technical optimization, regulatory engagement, and market development to ensure that scientific advances in flax genome editing translate efficiently into commercially viable outcomes.

Emerging technologies such as base editing, prime editing, compact CRISPR nucleases, virus-mediated delivery systems, and tissue culture-independent transformation strategies may further accelerate flax genome engineering by enabling precise and potentially transgene-free genetic modifications. The successful adaptation of these next-generation platforms to flax could substantially reduce genotype dependence, improve editing efficiency, and expand the range of agronomically relevant traits accessible to genome engineering.

In summary, the integration of advanced genome engineering technologies with trait-focused breeding strategies provides a comprehensive framework for unlocking the full potential of flax. Continued progress in precise editing systems, innovative delivery technologies, and genotype-independent transformation approaches is expected to accelerate both functional genomics research and practical cultivar development. Such combined approaches are expected to enhance its role as a sustainable dual-purpose crop for both fiber and nutritional oil production in future agricultural systems.

The eventual impact of these technologies will also depend on the evolution of regulatory frameworks governing genome-edited crops and on the adoption of internationally harmonized approaches for evaluating transgene-free edited plants.

## Figures and Tables

**Table 1:** Delivery strategies for plant genome engineering: Agrobacterium-, physical-, viral-, and nanoparticle-based approaches.

Method	Representative Species and Targets	Main Advantages	Main Limitations	Transformation/Editing Efficiency	Regeneration Capacity	DNA-Free Editing Possible	Heritable Edits	Cargo Capacity	Typical Off-Target Risk	References
***Agrobacterium*** **-mediated transformation**	Wheat (*Triticum aestivum*), soybean (*Glycine max*), cassava (*Manihot esculenta*), rice (*Oryza sativa*), cotton (*Gossypium hirsutum*), melon (*Cucumis melo*), apple (*Malus × domestica*), *Eucalyptus* spp., and legumes (pea, lentil, chickpea)	Low transgene copy number (typically single-copy integration), stable genomic integration, cost-effectiveness, high reproducibility, and the ability to transfer large DNA fragments (>30 kb). In many species, hypersensitive defense responses are minimized.	Strong genotype dependence, random integration that may affect endogenous genes or transgene expression, possible integration of vector backbone sequences, and the requirement for efficient tissue culture and regeneration systems.	Wheat (Fielder IE): 33–77.5%; wheat (ME): 0.65–7.5%; soybean (GiFT): 2–30%; melon (with DRs): 32.5–45.6%; rice: ~32%; cotton: ~60% positive regenerated plants; tobacco: ~65%; *Nicotiana glauca*: ~2.4%.	*Eucalyptus*: up to 80%; cotton (embryo axis): ~5.5%; soybean (MiniMax): ~30.2% shoot regeneration; chickpea: up to 77.7%; pea: 0.6–3.4%.	No (usually DNA integration)	High	>30 kb	Moderate–High—stable genomic integration can result in prolonged Cas expression, increasing the likelihood of off-target mutations	[53,54,55,56,57,58,59,60,61,62]
**Biolistic transformation (particle bombardment/gene gun)**	*Anthoceros agrestis*, sugarcane (*Saccharum officinarum*), onion (*Allium cepa*), *Nicotiana benthamiana*, wheat (*Triticum aestivum*), maize (*Zea mays*), rice (*Oryza sativa*)	Broad cargo delivery capability, including plasmid DNA, linear DNA, RNA, proteins, and ribonucleoprotein (RNP) complexes; independence from host–pathogen interactions; suitability for organelle transformation; and relatively rapid implementation.	Risk of severe cellular and genomic damage, high transgene copy number leading to instability or gene silencing, high equipment costs, and variable transformation efficiency.	*A. agrestis*: 569 ± 268 transiently expressing cells per bombardment; onion: ~16% CRISPR editing efficiency; sugarcane: 68 PCR-confirmed transgenic lines recovered.	*A. agrestis*: stable lines obtained within 8–10 weeks (~6 lines per 0.2 g tissue); sugarcane: successful callus recovery and plant regeneration after selection.	Yes (RNP delivery)	High	Very high	Biolistic transformation (DNA constructs)—Moderate–High—often associated with multiple-copy integration and prolonged transgene expression; DNA-induced genomic rearrangements may also occur. Biolistic transformation (RNP delivery)—Low—transient activity of Cas–sgRNA complexes limits exposure time.	[63,64,65,66]
**PEG-mediated protoplast transfection**	Poplar (*Populus* spp.), cotton (*Gossypium* spp.), castor bean (*Ricinus communis*), passion fruit (*Passiflora edulis*), chickpea (*Cicer arietinum*), *Pinellia ternata*, black huckleberry (*Vaccinium membranaceum*), mango (*Mangifera indica*)	Simple, inexpensive, and broadly applicable method with high transformation efficiency. Supports co-transformation of multiple constructs and DNA-free genome editing through RNP delivery. Regeneration from single cells reduces chimerism.	PEG-induced cytotoxicity, possible interference with downstream analyses, and limited regeneration capacity in many recalcitrant species. High-copy-number integration may also occur.	Cotton: 80–84%; poplar: ~67%; passion fruit: 83%; black huckleberry: 75.1%; castor bean: 50.7%; chickpea: up to 63%; *P. ternata*: 79.25%; mango: 84.38%.	Regeneration remains a major bottleneck. Castor bean regeneration is generally limited to microcallus formation. Chickpea regeneration remains genotype dependent.	Yes (RNP delivery)	Depends on regeneration	High	PEG-mediated protoplast transfection (plasmid DNA) Moderate—transient but often strong expression of editing reagent PEG-mediated protoplast transfection (RNP delivery) Low—short intracellular persistence of RNPs minimizes off-target activity.	[67,68,69,70,71,72,73,74,75,76]
**Electroporation**	Soybean (*Glycine max*) protoplasts (GmCPR5), cabbage (*Brassica oleracea*) protoplasts (PDS1), wheat (*Triticum aestivum*) microspores, oil palm protoplasts	Enables DNA-free genome editing through RNP delivery, provides rapid and efficient nucleic acid uptake, avoids PEG-associated toxicity, and can achieve high editing efficiencies in optimized systems.	Cell viability may be reduced by high-voltage pulses; extensive optimization is often required for each species and cell type; specialized equipment is necessary.	Soybean: 2.1–8.1% InDel frequency; cabbage: 1.2–3.4%; wheat: ~2.2% mutation frequency; plant growth-promoting bacteria (PGPB): 10^5^–10^6^ CFU μg^−1^ DNA.	Regeneration is often constrained by species-specific recalcitrance and genotype dependence, although successful protocols have been reported for selected crops.	Yes (RNP delivery)	Depends on regeneration	Moderate–High	Electroporation (plasmid DNA)—Moderate—transient expression generally results in lower risk than stable transformation. Electroporation (RNP delivery)—Low—transient presence of editing machinery reduces off-target events.	[77,78]
**Viral Vectors**										
**Negative-sense RNA viruses (NSVs) (BYSMV, SYNV, TSWV)**	*Nicotiana benthamiana* (NbPDS, NbFucT, NbDCL2, NbRDR6, NbSGS3, NbBBLd, GFP), tobacco, tomato, pepper, peanut, ground cherry (PDS), wheat (TaPDS, TaSDN1)	Large cargo capacity, enabling delivery of complete CRISPR systems (Cas proteins and sgRNAs) as well as base editors. DNA-free and non-integrative.	Generally excluded from meristematic tissues, requiring regeneration for transmission to progeny. Biosafety concerns may arise from insect-mediated transmission.	TSWV infection rates can reach 100% in agroinoculated plants; SYNV and BYSMV support efficient somatic editing.	BYSMV: 7.4–8.0% edited tillers; TSWV (with ribavirin): 30–68.9% biallelic mutants among regenerants.	Yes	Low–moderate	SYNV~5 kb TSWV > 5 kb	Moderate—prolonged systemic expression may increase editing duration, although no DNA integration occurs.	[79,80,81]
**Positive-sense RNA viruses (PSVs) (TRV, BSMV)**	Wheat (TaPDS, TaGW2, TaGASR7, TaHRC, TaQ), barley (PDS), maize (PDS, ZmTMS5, HKT1), *Arabidopsis* (AtFWA, AtGL1, AtTT4), *N. benthamiana* (NbPDS, NbAG, NbPCNA, NbChlH), soybean (EPSPS, GmGW2), cotton (PDS)	Efficient systemic movement, high infection rates, and the ability to generate heritable edits without tissue culture.	Limited cargo capacity; most systems require transgenic Cas9-expressing plants.	BSMV infection rates in wheat frequently exceed 85%.	TRV-mediated editing: 65–100% heritable mutations; BSMV-mediated editing in wheat: 12.9–100% edited progeny.	Usually yes (if Cas9 already present)	High	TRV~1 kb BSMV~1–2 kb	Moderate—systemic spread and sustained sgRNA expression can extend editing activity.	[44,79,81,82,83,84]
**Geminivirus-based vectors (ssDNA viruses) (BeYDV, WDV, CaLCuV)**	Tomato (SlANT1, SlCRTISO, SlPSY1), potato (StALS1), tobacco (AtADH1, ALS), wheat and rice (Ubi locus, GFP, BFP), cassava (EPSPS)	Particularly effective for HDR, targeted gene insertion, and delivery of repair templates due to high nuclear replication levels.	Deconstructed replicons generally lack systemic movement. Viral DNA integration into the host genome may occur.	High-copy replication can increase editing efficiency by 10–100-fold compared with conventional T-DNA delivery systems.	Typically tissue-culture dependent; up to 25% of T_0_ plants may show targeted integration, while WDV-mediated knock-in frequencies can reach 19.4% in wheat.	No (DNA replicon)	Moderate–high	Moderate	High—high-copy replication and prolonged expression of CRISPR components may increase off-target editing frequency.	[85,86,87,88]
**Nanoparticle-mediated delivery (CNTs, carbon dots, mesoporous silica nanoparticles, lipid nanoparticles, magnetic nanoparticles)**	*Nicotiana benthamiana*, wheat (*Triticum aestivum*), cotton (*Gossypium hirsutum*), maize (*Zea mays*), lettuce (*Lactuca sativa*), arugula (*Eruca sativa*)	DNA-, RNA-, protein-, and RNP-delivery without genomic integration; minimal tissue damage; potential species-independent delivery; suitable for transient expression and DNA-free editing	Still largely experimental; delivery efficiency and reproducibility vary among species; limited whole-plant regeneration data; nanoparticle toxicity remains under investigation	Typically 10–80% transient expression depending on nanoparticle type and species; stable editing frequencies remain highly variable	Usually not regeneration-dependent for transient expression; regeneration protocols for edited plants remain limited	Yes	Currently low–moderate; heritable editing	High	Low (particularly with RNP delivery)	[45,89,90,91]
**In planta transformation/de novo meristem induction (BBM/WUS, GRF-GIF, Fast-TrACC, Cut-Dip-Budding**	Wheat (*Triticum aestivum*), maize (*Zea mays*), sorghum (*Sorghum bicolor*), soybean (*Glycine max*), tomato (*Solanum lycopersicum*), tobacco (*Nicotiana tabacum*	Avoids conventional tissue culture; reduces genotype dependence; enables direct recovery of edited shoots; suitable for recalcitrant crops	Editing efficiency still species dependent; developmental regulators may induce abnormalities; protocols are not yet universally transferable	Frequently 1–30% edited shoots depending on species and construct	High; regeneration occurs directly through induced meristems	Usually No (*Agrobacterium*-based systems dominate), but potentially yes with RNP-compatible approaches	High	High (>30 kb when *Agrobacterium*-based)	Moderate	[35,36,37,38]

BSMV, *Barley stripe mosaic virus*; BYSMV, *Barley yellow striate mosaic virus*; CaLCuV, *Cabbage leaf curl virus*; HDR, homology-directed repair; NSV, negative-sense RNA virus; PSV, positive-sense RNA virus; RNP, ribonucleoprotein; SYNV, *Sonchus yellow net virus*; TRV, *Tobacco rattle virus*; TSWV, *Tomato spotted wilt virus*; WDV, *Wheat dwarf virus*; BeYDV, *Bean yellow dwarf virus*.

**Table 2:** Genome Editing Systems in Plants: A Comparative Overview of CRISPR-Based Tools, Base Editors, and Prime Editors.

Editing System	PAM	Plant Species	Key Features	Target Loci	Objective	Editing Efficiency	Efficiency Assessment	Delivery/Transformation	Explant Type	Regeneration Outcome	Reference
LrCas9-CBE (PmCDA1)	5′-NGAAA-3′	Rice (*Oryza sativa*), Wheat (*Triticum aestivum*)	High-fidelity CBE; expands A/T-rich target space	*OsDEP1*, *OsPDS*	Expand base editing scope	Up to 90.5% (T0 rice)	Deep sequencing, Hi-TOM	Agrobacterium-mediated; PEG (protoplasts)	Embryonic calli; leaf protoplasts	High (rice), moderate (wheat) protoplast efficiency	[128]
FrCas9	5′-NNTA-3′	Rice	Recognizes 16 NNTA variants; promoter targeting tool	*OsWX*, *OsSWEET13*, *OsPDS*	Fine-tune promoter activity	31.3–35.3%	NGS, Sanger	Agrobacterium (EHA105)	Seed-derived embryonic calli	>300 resistant calli	[138]
SpCas9-NG	5′-NGD-3′/5′-RGC-3′	Soybean (*Glycine max*)	Broad PAM compatibility; miRNA targeting	miR156a, miR172a, *NARK*	Expand targetable loci	~5.3% (NGA loci)	Deep amplicon sequencing	Agrobacterium rhizogenes (hairy roots)	Hypocotyls	Not applicable	[129]
PE3	5′-NGG-3′	Poplar (Populus hybrid 84 K)	Precise substitutions and small indels	*PagPDS*, *PagYUC4*, *PagSHR*	Establish prime editing in trees	3.6–22.2%	NGS, Sanger	Agrobacterium-mediated	Leaf disk calli	Low, unstable regeneration	[139]
zCas9i (multi-gRNA)	5′-NGG-3′	*Arabidopsis thaliana*	Intron-optimized; multiplex deletions	*AtWRKY30*	Large chromosomal deletions	6.5–12.5%	PCR, PacBio	Floral dip	Floral tissues	High regeneration efficiency	[140]
rXRCC1-CGBE/UNG-CGBE	5′-NGG-3′/SpRY	Rice, Tomato, Poplar	C-to-G base editors with BER proteins	*OsALS*, *SlAGO7*, *PtPDS1*	Enable C → G transversions	Up to 38% (rice)	NGS, Hi-TOM	*Agrobacterium*; PEG	Embryonic calli; protoplasts	192 T0 poplar lines	[133]
A3A/CDA1 CBE (optimized)	5′-NGG-3′	Potato (*Solanum tuberosum*)	Enhanced CBE via native promoter	*StGBSS1*	Improve editing efficiency	Up to 43%	Sanger, EditR, IDAA	PEG-mediated	Leaf protoplasts	Uniform regenerated lines	[135]
SaCas9	5′-NNGRRT-3′	Soybean	Compact nuclease (delivery advantage)	*GmFT2a*, *GmFT5a*	Expand genomic targeting	34.5–73.3%	CAPS, Sanger	*Agrobacterium rhizogenes*	Hypocotyls	Hairy root system only	[141]
Cas9-HDR (GATIPS)	5′-NGG-3′	Rice	HDR-mediated precise insertion (1182 bp)	*OsEPSPS*	Glyphosate resistance	~15%	PCR, Southern blot	Biolistic (gene gun)	Embryonic calli	Successful regeneration	[142]
Cas9-PaU6 (spruce-optimized)	5′-NGG-3′	White spruce (*Picea glauca*)	First stable conifer editing	*PgDXS1*	Overcome conifer barriers	Up to 28%	Sanger, phenotype	*Agrobacterium*-mediated	Embryogenic callus	>2000 somatic embryos regenerated	[143]
Mb3Cas12a	5′-TTTN-3′	Rice	Temperature-sensitive Cas12a variant	*OsPDS*	Evaluate temperature dependence	27.4% at 37 °C	NGS, phenotype	Biolistic	Embryonic callus	Efficient shoot regeneration	[112]
Fanghui Casδ	5′-RYR-3′	Maize	Engineered high-specificity compact nuclease	*TS4*, *PSY1*	Improve Casδ activity	Up to 84%	Sanger, NGS	*Agrobacterium*	Callus/embryos	Not specified	[119]
PhieABEs	5′-NNN-3′	Rice	PAM-less ABE; wide editing window	*OsSPL14*, *OsIAA13*	High-efficiency adenine editing	Up to 100% (sites)	NGS, Hi-TOM	*Agrobacterium*	Callus-derived plants	No chimerism detected	[132]
CRISPR-FrCas9	5′-NNTA-3′	Rice	TA-site targeting system	*OsGn1a*, *OsGS3*	Functional mutagenesis	35–80.7%	Sanger	*Agrobacterium*	Callus	Not specified	[129]
en4Cas12j-8	5′-TTN-3′	Soybean, Rice	Hypercompact nuclease (1016 aa)	*GmPDS1*, *OsDEP1*	Improve compact system efficiency	Up to 91.9%	NGS	*Agrobacterium*	Hairy roots/T0 plants	Not specified	[122]
PhieDBEs	5′-NGD-3′	Rice	Dual A/C base editing	*IPA1*, *TGW6*	Simultaneous conversions	Up to 95.2%	NGS, Hi-TOM	*Agrobacterium*	Embryonic calli	Not specified	[144]
NM-PE	5′-NNN-3′ (SpRY)	Rice	MMEJ-assisted prime editing	*OsMPK3*, *OsMSP1*	Epitope tagging	>2.5× improvement	Deep sequencing	*Agrobacterium*	Transgenic plants	Not specified	[109]
rPE20	5′-NGG-3′	Rice	Long-fragment prime editing (up to 66 bp)	*OsCPK3*, *OsALS*	Improve insertion efficiency	Up to 26%	Deep sequencing	*Agrobacterium*	T0 plants	Not specified	[145]
TRV-VIGE	5′-NGG-3′	*Nicotiana attenuata*	Virus-based, tissue-culture-free editing	*NaPDS*, *NaCHAL2*	Heritable editing without regeneration	2–6%	Deep sequencing	TRV agroinfiltration	Meristematic tissue	M1 seeds obtained	[146]
pECNUS4	5′-NGV-3′	*Arabidopsis thaliana*	ABE with selectable marker (FastRed)	PDS3, DM3	Transgene-free selection	Up to 81%	Amplicon sequencing	Floral dip	Floral tissues	High efficiency	[147]
TRSV-VIGE	5′-NGG-3′	*Nicotiana benthamiana*	Shoot meristem viral editing system	NbPDS, NbAG	Germline editing via virus	0.8–13.2%	CAPS, sequencing	TRSV inoculation	Shoot meristem	Lateral shoot recovery	[148]
CoCas9	Not specified	Citrus	Compact human-microbiome nuclease	*CsLOB1*	Alternative Cas9 variant	0.9 ± 0.2	PCR, sequencing	*Agrobacterium*-mediated	Transgenic plants	Not specified	[124]

Abbreviations: Cas9, CRISPR-associated protein 9; CBE, cytosine base editor; ABE, adenine base editor; PE, prime editing; HDR, homology-directed repair; MMEJ, microhomology-mediated end joining; VIGE, virus-induced genome editing; NG, protospacer adjacent motif variants; T0, first-generation transgenic plants; NGS, next-generation sequencing; CAPS, cleaved amplified polymorphic sequences; IDAA, indel detection by amplicon analysis. Notes: Editing efficiency values represent reported maximum or range in T0 or early-generation plants unless otherwise stated. “Not specified” indicates unavailable regeneration or efficiency data in the original study.

**Table 3:** Representative SSR and SNP molecular markers in *Linum usitatissimum* L. and their applications in genetic diversity analysis, linkage mapping, and trait association studies.

Marker Type	Marker Set/Resource	Marker Characteristics	Target Locus/Genomic Source	Main Application	Reference
SSR	BES-SSR markers	673 polymorphic SSR primer pairs	BAC-end sequences (BES)	Genetic diversity analysis, linkage mapping, QTL mapping, physical map anchoring	[158]
SSR	EST-SSR markers	145 polymorphic SSR primer pairs	Expressed Sequence Tags (ESTs)	Functional marker development, diversity studies, linkage mapping	[158]
SSR	Flax SSR collection	818 newly developed polymorphic SSRs (869 loci)	BES and EST sequences	Association mapping, QTL analysis, genome anchoring	[158]
SSR	Consensus map SSR markers	770 mapped SSR loci	Whole flax genome linkage groups	Construction of integrated genetic and physical maps	[159]
Gene-based markers	fad2A	Gene-specific marker mapped on linkage map	LuFAD2A	Fatty-acid biosynthesis studies	[159]
Gene-based markers	fad2B	Gene-specific marker mapped on linkage map	LuFAD2B	Fatty-acid biosynthesis studies	[159]
Gene-based markers	fad3A	Gene-specific marker mapped on linkage map	LuFAD3A	Fatty-acid composition studies	[159]
Gene-based markers	fad3B	Gene-specific marker mapped on linkage map	LuFAD3B	Fatty-acid composition studies	[159]
Gene-based markers	dgatA	Gene-specific marker mapped on linkage map	LuDGATA	Triacylglycerol biosynthesis studies	[159]
Gene-based markers	dgatB	Gene-specific marker mapped on linkage map	LuDGATB	Oil accumulation studies	[159]
Gene-based markers	ysc1	Gene-specific marker mapped on linkage map	YSC1 locus	Seed colour studies	[159]
SNP	Genome-wide SNP resource	Thousands of putative SNPs discovered by reduced-representation sequencing	Genome-wide	High-density mapping, association studies, genomic analyses	[160]

**Table 4:** Regeneration systems used in flax (*Linum usitatissimum* L.) genome editing, including explant type, culture conditions, and efficiency.

Explant Type	Culture Medium	Hormonal Composition (Auxin/Cytokinin)	Regeneration Pathway	Time to Regeneration	Efficiency (%)	Notes/Limitations	References
Hypocotyl segments	MS medium	2,4-D (0.5–2.0 mg/L) for callus induction; TDZ (0.1–1.0 mg/L) or BAP (0.5–2.0 mg/L) for shoot induction	Indirect organogenesis via callus	6–12 weeks	10–60%	Strong genotype dependence; frequent browning and oxidative stress during callus phase	[174,179]
Cotyledon explants	MS or B5 medium	BAP (0.5–2.0 mg/L) + NAA (0.1–0.5 mg/L)	Direct organogenesis	4–8 weeks	20–70%	Higher regeneration stability than hypocotyl-derived callus; still genotype-dependent	[174]
Immature embryos	MS medium	Low auxin (NAA 0.1–0.3 mg/L) + BAP (1.0–2.0 mg/L)	Somatic embryogenesis/organogenesis	5–9 weeks	30–75%	Higher efficiency, reduced somaclonal variation compared to callus systems	[175]
Leaf discs	MS medium	TDZ (0.2–1.0 mg/L) + NAA (0.1–0.3 mg/L)	Indirect organogenesis	6–10 weeks	15–55%	Tissue browning and oxidative stress common; requires antioxidant optimization	[180]
Apical meristem/*in planta*	No callus medium required (*in planta* system)	None or minimal hormone supplementation	Direct shoot regeneration	3–6 weeks	25–65%	Reduces somaclonal variation; lower transformation efficiency but more stable lines	[169]
Protoplasts (leaf mesophyll)	W5/K3-derived regeneration medium	Initial hormone-free culture; later 2,4-D (0.5–1.5 mg/L) + BAP (0.5–1.0 mg/L)	Protoplast → callus → shoot regeneration	8–16 weeks	5–30%	Low regeneration efficiency; high osmotic stress sensitivity; strong genotype dependence	[181]
Cell suspension-derived callus	MS medium	2,4-D (1–2 mg/L) + kinetin (0.5–1 mg/L)	Somatic embryogenesis	7–14 weeks	10–40%	Useful for transformation studies but prone to somaclonal variation and instability	[170]

## Data Availability

No new data were created or analyzed in this study. Data sharing is not applicable to this article.
